# Supplementary material for: Content-rich biological network constructed by mining PubMed abstracts
Source: BMC Bioinformatics. 2004 Oct 8;5:147. doi: 10.1186/1471-2105-5-147 (PMC528731; doi:10.1186/1471-2105-5-147)
Supplement: Additional File 2 — The original results of the above study (non-essential files are deleted to keep the file size under the limit set by BMC bioinformatics). [file 1471-2105-5-147-S2.bz2 › chilibotAdditionalFile2/dip05/665ID10428814E11059/html/TNFRSF5_TRAF3.html]

 


 **TNFRSF5** and **TRAF3** 
  
Found 115 abstracts in PubMed, retrieved 05.  
 

 What does Google say? 
 PDF only 
| .edu only 

---

**Interactive relationship** (e.g. stimulation, inhibition, etc)

**Neutral relationship**- Latent membrane protein 1 LMP1  [ **TRAF3** ]  is an Epstein Barr virus EBV encoded, ligand independent receptor that mimics CD40  [ **TNFRSF5** ] .  Ref: 12805217 EMBO J, 2003

**Non-interactive relationship** (e.g. studied together, co-existance, homology, etc.)

- The fusion of the transmembrane domain of LMP1  [ **TRAF3** ]  to signaling domains of CD40  [ **TNFRSF5** ] , TNF R1 and Fas activates their signaling.  Ref: 12805217 EMBO J, 2003
- Additionally, fusion constructs of the transmembrane domain of LMP1  [ **TRAF3** ]  and the intracellular signaling domain of CD40  [ **TNFRSF5** ] , TNF R1, and TNF R2 likewise transactivated the CD83 promoter via NF kappaB.  Ref: 12857898 J VirolJ Virol, 2003
- we demonstrate that a fusion of LMP1  [ **TRAF3** ]  with CD40  [ **TNFRSF5** ]  recruits TRAF2 intracellularly.  Ref: 12805217 EMBO J, 2003
- Furthermore, resistin upregulates adhesion molecules and chemokines and downregulates TRAF 3  [ **TRAF3** ] , an inhibitor of CD40  [ **TNFRSF5** ]  ligand signaling.  Ref: 12874180 CirculationCirculation, 2003
- The latent membrane protein 1 LMP 1  [ **TRAF3** ]  oncoprotein of Epstein Barr virus EBV is a constitutively active, CD40  [ **TNFRSF5** ]  like cell surface signaling protein essential for EBV mediated human B cell immortalization.  Ref: 12610149 J Virol, 2003
- Like ligand activated CD40  [ **TNFRSF5** ] , LMP 1  [ **TRAF3** ]  activates NF kappaB and Jun kinase signaling pathways via binding, as a constitutive oligomer, to tumor necrosis factor receptor associated factors TRAFs.  Ref: 12610149 J Virol, 2003
- Taken together, these data further our understanding of the function of LMP1  [ **TRAF3** ]  in epithelial cells and suggest that LMP1  [ **TRAF3** ] , similar to its mammalian homolog CD40  [ **TNFRSF5** ] , can exert opposing effects on cell survival depending on the nature of the apoptosis trigger.  Ref: 12504573 Virology, 2002
- The effects of resistin on expression of vascular cell adhesion molecule VCAM 1 and monocyte chemoattractant chemokine MCP 1 were studied in addition to CD40  [ **TNFRSF5** ]  receptor, CD40  [ **TNFRSF5** ]  ligand induced MCP 1 expression, and tumor necrosis factor receptor associated factor 3  **TRAF3** , an inhibitor of CD40  [ **TNFRSF5** ]  signaling.  Ref: 12874180 CirculationCirculation, 2002
